# Supplementary material for: Are carbon emissions trading and green financial instruments synergistic? -Comprehensive quantitative research based on content analysis
Source: PLoS One. 2024 Mar 7;19(3):e0298601. doi: 10.1371/journal.pone.0298601 (PMC10919601; doi:10.1371/journal.pone.0298601)
Supplement: S1 Data — (ZIP) [file pone.0298601.s001.zip › Data/地级市Z_F工作报告.zip!/╜¡╬≈/╛┼╜¡╩╨/└┤╫╘╣1⁄2╓┌║┼í╛┬φ┐╦╩2╛▌═°í┐_2013.docx]

各位代表：

　　现在，我代表市人民政府，向大会报告工作，请予审议，并请各位市政协委员和列席会议的同志提出意见。

　　一、2012年工作回顾

　　过去的一年，在省委、省政府和市委的正确领导下，全市上下以科学发展观为指导，以党的十八大胜利召开为强大动力，坚持“两区互动、强工兴城”不动摇，深入推进鄱阳湖生态经济区建设和沿江开放开发，着力抓项目、扩固投、稳运行、惠民生，有效克服了宏观经济环境的不利影响，全面完成了年初确定的各项任务，又奋力迈出了加快赶超的坚实步伐。

　　（一）赶超发展步伐持续加快，财政收入跃上200亿元新台阶。全市实现生产总值1420.1亿元，同比增长12%。财政总收入达到220.86亿元，净增59.06亿元，增长36.5%；地方一般预算收入141.87亿元，增长40.4%；财政总收入占GDP比重提高2.7个百分点，达到15.6%；县级财政总收入完成176.77亿元，9个过10亿元，5个过15亿元，九江经济技术开发区过20亿元。全市社会消费品零售总额382.7亿元，增长16.1%。金融机构贷款余额突破1000亿元。外贸出口35.3亿美元，增长34.3%。城镇居民人均可支配收入20330元，农民人均纯收入7785元，分别增长13.5%、14.9%；居民消费价格总指数102.8%。

　　（二）工业经济逆势上扬，规模以上主营业务收入突破3000亿元。规模以上工业主营业务收入增长28.6%，净增817亿元；增加值621.3亿元，增长14.6%；利税总额270亿元，增长38.4%；工业固投948亿元，增长30.1%，占全市固投的70%以上；工业用电量76.2亿度，增长10.4%。通过落实小微企业帮扶政策，积极组织政银企对接，有效开展“银园保”合作，全力帮助企业破难题、促生产、保增长，工业经济低开高走，对全市经济的主导作用更加凸显。通过推进产业招商，打造龙头，完善链条，十大产业集群实现主营业务收入2453.2亿元，占全市比重超过80%。通过帮扶企业外拓市场，内强管理，技改扩能，新增规模以上工业企业95户，达846户；新增利税过亿元企业18户，达37户；新增主营业务收入过亿元企业92户，达588户，过10亿元的新增6户，达31户，九江石化突破300亿元，旭阳雷迪等一批企业过50亿元。通过推进企业技术创新，电子信息、新材料等高新技术产业发展迅速，实现主营业务收入620亿元，增长35%，增加值132亿元，增长20%，高新技术产业占GDP比重提高2.1个百分点。通过加大投入，完善功能，优化服务，园区新增开发面积20平方公里，达110平方公里，新增标准厂房30万平方米、园区公租房20万平方米；主营业务收入突破2000亿元，新增百亿园区2个，九江开发区一年净增110亿元，九江出口加工区出口总额继续保持中部地区第一，永修云山经济开发区、德安县工业园获批省级重点园区。

　　（三）兴城势头继续保持，城建投入超过500亿元。全市实施城建项目926个、完成投资522亿元，城镇化率提高1.9个百分点。中心城区实施城建项目160个，竣工34个，在建75个，完成投资190亿元。《大九江城市群空间发展战略规划》和《九江市城市综合交通体系规划》通过论证评审，绕城高速顺利开工，老九湖路改造完工，大九江区间通道加快成型，“四县一山”与中心城区发展规划、资源配置、产业布局、公共服务、基础设施、社会管理的一体化进程明显加快。八里湖新区控制性详规和土地利用规划编制完成，“五个一批”107个重点项目快速推进，全年完成政府性投资50亿元，带动社会投资100亿元；市博物馆、八里湖大桥、前进西大道、环湖三路及景观桥、海韵沙滩、蛟滩水利综合整治等30个项目建成投运，市民服务中心、文化艺术中心、九龙山公园、中航城等47个项目加快推进，寻阳古城景区、西组团路网等30个项目启动实施，为“五年基本建成生态新区”奠定了坚实基础。通过创新机制，下放权限，区内运作，全面加快老城区改造，浔阳区战备路、化工厂、琵琶亭等9个片区改造加快推进，德化路、花果园等7处安置房启动建设；庐山区城南核心商业区快速推进，十里南大道、莲花大道、前进南路、九莲北路、九莲南路等竣工通车；九江开发区长江大道、九瑞大道、八里湖北岸大堤等改造项目全面完工；积极实施中心城区大水系综合治理工程，白水湖及浔南片区、濂溪片区截污工程启动建设，城区水系连通工程前期工作有序推进。县城和集镇建设加快推进，各县共实施城建项目766个，竣工457个，完成投资332亿元，一批规划科学、配套齐全、环境优美、特色鲜明的县城新区初具规模。成功创建国家园林城市、全国无障碍建设先进城市，实现全国双拥模范城市创建“四连冠”，第四届省级文明城市创建通过复检；积极开展“两声三乱”等综合整治，规范城区沿江砂石站管理，创新城建项目管护模式，建立建筑工地数字化监控平台，城市管理水平、市容环境、文明程度进一步提升。

　　（四）沿江开放开发势头良好，完成项目投资900亿元。沿江开放开发上升为省级战略，省委、省政府出台“两个意见”，发布“1＋5”规划，建立省级调度机制，安排总用地规模113平方公里；省直相关部门出台扶持政策，加大财税、人才、资金、用地、环保等支持力度，为沿江开放开发注入了新的动力。成立市沿江开放开发领导小组、四大板块协调小组和市沿江开放开发办公室，健全调度、倒逼、督查、服务等工作机制，设立3000万元专项资金，争取省财政基础设施扶助资金1.5亿元；市直各部门、驻市各单位、沿江各县（市、区）力量向沿江集合，要素向沿江集聚，凝成了强大的开发合力，四大板块成为客商投资的热点和产业集聚的平台。沿江区域签约、开工、竣工各类项目45批次，开工316个，竣工86个；固定资产投资、实际利用外资、出口总额和规模以上工业增加值、主营业务收入占全市比重均超过60%。“双十工程”加快推进，城东城西疏港通道、彭湖沿江大道（湖口段）等建成通车，赤码大道启动建设，四大板块路网基本成型；城西港列入九江港口岸扩大开放范围获国务院批准，电子口岸平台检港系统上线运行，九江港至洋山港国际集装箱运输支线、出口烟花鞭炮水运通道开通运行；新建、续建3000吨级以上泊位22个，取缔非法码头（泊位）13个，整合搬迁45个；沿江港口货物吞吐量超过4800万吨，集装箱吞吐量16万标箱；新引进10亿元以上重大产业项目76个，总投资936亿元，开工33个，完成投资136亿元。

　　（五）“三农”工作扎实推进，粮食生产实现“九连增”。农业产业化步伐明显加快，大宗农产品稳产增产，粮食总产158.3万吨；引进亿元以上项目52个，神州通油茶等项目进展顺利，新增规模以上企业46家，销售收入过10亿元的达6家，江西省新光山水开发有限公司被评为国家级龙头企业；新增省级现代农业示范区3个，创建部级万亩水稻高产示范片55个；“仙客来”、“绿冬丝科”荣获中国驰名商标；我市在省花博会、中国绿色食品博览会上获多项金奖；新增农民专业合作社410家、“农超对接”点55个。农业科技创新力度不断加大，新增省级农业科技示范区3个、示范基地3个，实施科技开发项目150个，引进推广农业新技术、新品种390项；建设信息化乡镇50个、农村信息服务站500个。农村基础设施建设扎实推进，81座重点小（Ⅰ）型、176座重点小（Ⅱ）型水库除险加固和8条中小河流治理防洪工程全面完成，解决30万农村人口的安全饮水问题。新增、恢复灌溉面积9万亩，改善灌溉面积18万亩。落实农业“四项补贴”资金3.4亿元，建设高标准农田7.2万亩，改造中低产田6万亩，完成水土流失综合治理24万亩。市财政安排专项资金3000万元大力推进和谐秀美乡村建设，高速公路和环庐山沿线村庄整治效果明显，完成通道绿化200公里。新增改造农村公路1016公里。934个新农村省级建设点改水、改路、改厕任务全面完成，培育产业村661个、产业大户2198户。继续实施垃圾处理“445”模式，农村清洁工程效果明显。共青城4个“城乡一体化新区”基本建成。

　　（六）重大项目加快实施，固定资产投资达到1300亿元。全市实施亿元以上重大项目85个，其中10亿元以上27个，纳入省重点调度的55个，争取各类专项资金20亿元；引进省外5000万元以上项目175个，实际进资360亿元，其中亿元以上项目80个，实际进资181亿元；实际利用外资9.9亿美元，增长27.9%。一批重大产业项目快速推进，投资50亿元以上的理文造纸、共晶光伏，10亿元以上的中盛陶瓷、京九电源，5亿元以上的金鼎钨钼矿、绿晶光电、银河表计等项目全面建成，部分投产；投资100亿元的石化油品质量升级工程、恒生大化纤，50亿元以上的九宏新材料、理文化工，20亿元以上的龙达二期、攀森二期、青岛啤酒、亚泥五六期等项目快速推进；投资100亿元的中通金域云计算中心，投资30亿元以上的德鑫纺织二期、金旺国际物流园等项目开工建设；神华煤电、千万吨级优质钢、杭氧工业气体等项目启动前期。一批重点基础设施项目快速推进，巾南线、修万线、316国道（瑞昌段）、德白线、定龙线等公路竣工通车；全年新增装机容量69.3万千瓦，红光、九化、通江岭等输变电工程建成投运；九江长江公路大桥、昌九高速（通远段）改扩建、都中公路改造、辽南公路改造、105国道（九江段）改造、九电四期、大唐永修风电、华电分布式能源和马回岭、蔡岭输变电等项目快速推进；彭湖板块公用码头启动建设，赤码板块理文公用码头基本建成；九江机场航班增至每周24个。一批重要社会事业项目快速推进，中华贤母文化园一期、市一医院康复保健楼等项目竣工；双峰小学庐山区校区、九江学院八里湖校区等项目快速推进；九江职业大学、九江职业技术学院新校区，九江小学八里湖校区，开发区综合医院等项目开工建设。

　　（七）社会事业全面进步，财政支出民生资金170亿元。

　　省定民生工程和市定十件民生实事全面完成。整合资金110.6亿元，新开工保障性住房8.5万套，新竣工9.1万套，为1.6万户发放租赁补贴。新增财政扶贫资金9500万元，整合扶贫和移民资金4.45亿元，整村推进395个贫困村扶贫开发，惠及15万户、64万人。新增城镇就业6.4万人，城镇就业率98.5%；新增转移农村劳动力6.1万人，发放小额贷款8.2亿元，“零就业”家庭实现动态清零；城乡低保、抚恤优待、农村五保和城镇“三无”对象供养、孤儿养育等标准进一步提高，医疗救助27万人次；筹措各类社会救助资金9.6亿元，发放低保金5亿元，惠及城乡困难家庭7.4万户、27万人；城镇职工基本养老保险参保缴费47.9万人，城镇基本医疗保险参保缴费132.7万人，城乡居民社会养老保险142.5万人，被征地农民养老保险4.6万人，失业保险33.5万人，工伤保险46万人；“光明·微笑”工程扎实推进，继续对儿童白血病、先天性心脏病和困难尿毒症患者、晚血病人实施免费救治；深山区、地质灾害区移民搬迁8968人，农村重点污染区域整体搬迁移民2549人，农村危房改造5000户；完成中心城区3家农贸市场标准化升级改造，新扩城郊蔬菜基地6000亩；强化质量安全监管，食品、药品、农资和“侵权假冒”等专项整治成效明显。全面落实信访接待日和领导包案化解制度，信访形势总体平稳；全面开展安全生产“打非治违”专项行动，推进安全生产标准化建设，安全事故发生率和安全事故死亡人数双下降；扎实推进“六五”普法，深入开展社会管理综合治理，依法打击各类违法犯罪，切实维护国家安全和社会稳定，公众安全感位居全省前列。新增国家高新技术企业11家、省级以上科研基地5个，恒盛科技（创业）园升级为国家级高新技术创业中心，争取省部级科技计划项目120个，专利授权560件；各类教育全面发展，教育经费支出占GDP比重首次超过4%，中小学校舍三年规划改造项目全部完成，补充农村学校教师1200名；一批重点文化设施建成开放，农家书屋实现行政村全覆盖，申报省级“非遗”名录14个，市、县、乡、村四级公共文化服务体系基本形成；新农合参合率98%，补偿资金5.4亿元，新增全国示范社区卫生服务中心2个，市中医院通过三甲评审；成功举办市第十一届运动会暨全民健身运动会，在省第四届全民健身运动会上再创佳绩；人口自然增长率7.25‰，提前两年实现农村计生纯女户社会养老保险全覆盖；在全省率先推行村（居）“两委”选举新模式，农村社区建设经验在全国推广。统计、审计、海关、检验检疫、气象、水文、盐业、通讯、邮政、无线电管理、档案、保密、编制、地方志、对台、外事侨务、民族宗教等工作得到新加强；国防动员、民兵预备役、人民防空、防震减灾、消防等工作取得新成绩；工会、共青团、妇女儿童、老龄、残疾人、红十字和慈善等事业实现新发展。

　　（八）更加注重扬优挖潜，发展活力明显增强。旅游强市建设迈出新步伐，全年接待游客突破4000万人次，旅游总收入突破300亿元，继续保持全省领先。设立1000万元旅游产业发展基金，旅游城市形象广告首次在央视播出，首届国际名湖友好交流大会顺利召开，“中小学教材景观旅游示范基地”启动建设，推出“春江花月夜”大型演艺剧目；石钟山创建4A级景区通过验收；庐山行政服务中心正式下迁，“庐山如意卡”开创全国景区会员制先河；中信庐山西海度假区、庐山新城、庐山建成区道路改造、东林大佛游览区等项目顺利推进；庐山西海管理体制进一步理顺。金融、保险、证券业加快发展，小额贷款公司实现县域全覆盖，成功发行二期企业债券20亿元。现代综合大市场、21世纪家居中心、大润发广场等项目顺利推进，禧徕乐商贸中心、联盛九龙广场、京九国际商贸城和一批商会总部楼宇项目启动建设；“万村千乡市场工程”扎实推进，完成488家农家店信息化改造。造林绿化34万亩，松材线虫病得到有效防治，鄱阳湖湿地保护和越冬候鸟保护工作进一步加强；柘林湖被列入“十二五”水质良好湖泊生态环境保护试点，全面清理库湾网箱养殖。新增省级生态乡镇11个、生态村17个；积极开展环保专项行动，重点耗能企业和公共机关节能有效加强，省政府下达的节能减排任务全面完成。非工口七大系统112户国有企业改革全面完成，安置职工8.3万人。浔阳区被列为省级服务业综合改革试点区。文化体制改革成效显著，荣获“全国文化体制改革工作先进地区”称号；医药卫生体制改革全面推进，实现国家基本药物制度乡、村两级医疗机构全覆盖；在全国率先推行新农合“先看病，后付费”诊疗服务模式，并实现公立医疗机构全覆盖；进一步规范鄱阳湖采砂管理，实现经济、社会和生态效益的有机统一。事业单位、财政、户籍、行政管理、国资监管和投融资体制等改革全面推进。

　　（九）政府自身建设不断加强，发展环境进一步优化。按照“核心党委、高效政府”的定位，坚决落实市委决策部署，强化操作，高效执行，优化服务，提升效能。主动接受市人大及其常委会的法律监督，自觉接受市政协的民主监督，充分听取各民主党派、工商联的意见建议，加强与工会、共青团、妇联等群众团体联系。办理人大代表建议201件、政协提案335件，研究处理落实市人大常委会审议意见建议9件。建立健全协调、推进、督查、奖惩等工作机制，强化项目建设政策、要素、责任、环境等保障措施，有效破解资金、土地等一系列发展难题。集中开展以“五治”为主要内容的干部作风突出问题整治活动，通过媒体问政、下访问效、跟踪问责，干部作风进一步好转。深入推进项目信息公开体系和“四中心一平台”建设，推行项目审批代办制和重大项目审批“绿色通道”，精简非行政许可审批项目63项。拓宽群众诉求渠道，民声直通车受理群众诉求7850件，办结率99.6%。深入开展政风行风评议，加大涉及群众切身利益和工程建设领域突出问题整治力度，全面落实党风廉政建设责任制，深入推进风险岗位廉能管理，权力运行监督机制和反腐倡廉惩防体系进一步健全。

　　各位代表，回顾一年来的工作，我们紧紧围绕赶超发展的目标，牢牢把握强工兴城的重点，抢抓机遇，应对挑战，强化操作，攻坚破难，巩固和发展了市委九届七次全会以来的好氛围、好势头、好局面，在“三年财政翻番”的基础上，继续迈出了“五年全面进位”的坚实步伐。这些成绩的取得，离不开省委、省政府和市委的正确领导，离不开市人大、市政协和社会各界的关心支持，离不开全市人民的共同努力。在此，我代表市人民政府，向辛勤劳动在各领域的全市人民，向大力支持政府工作的各位人大代表、政协委员，向离退休老同志，向各民主党派、工商联、人民团体和社会各界人士，向驻浔部队和武警官兵，向中央和省驻浔各单位，向所有关心、支持、参与九江建设和发展的各界朋友，表示衷心的感谢并致以崇高的敬意！

　　各位代表，回顾几年来的赶超历程，我们更深刻地体会到：上级关怀重视、领导高位推动，是我们加快赶超的强大动力；坚持两区互动、聚力强工兴城，是我们加快赶超的根本出路；市委坚强领导、班子共识共为，是我们加快赶超的有力保障；发展依靠人民、群众支持参与，是我们加快赶超的力量源泉；强化务实操作、全力攻坚破难，是我们加快赶超的关键所在。

　　各位代表，要实现赶超发展的既定目标，我们必须清醒地认识到：宏观经济环境的不确定、不稳定因素仍然存在；区域竞争更加激烈，九江争得应有地位的责任仍然重大；总量不大、实力不强、发展不足仍是九江当前和今后一个时期的主要矛盾，推进工业化、信息化、城镇化和农业现代化的担子仍然繁重；群众就业、住房、增收等方面还存在较大压力，改善民生、建成小康的任务仍然艰巨；影响和谐稳定的因素还不同程度存在，机关效能还有待进一步提升，优化环境依然需要加大力度。对这些困难和问题，我们将采取更加有力的措施，认真加以解决。

　　二、2013年工作安排

　　今年是全面贯彻落实党的十八大精神的开局之年，是实施“十二五”规划的关键之年，是实现五年全面进位的冲刺之年。尽管当前宏观经济环境仍然错综复杂、充满变数，尽管不平衡、不协调、不可持续问题依然突出，尽管企业生产经营成本上升和创新能力不足的问题并存，尽管微观运行的困难仍然存在，经济下行的压力仍然较大。但是，随着国家把沿江中部地区作为新形势下扩大开放、拉动内需的战略支点和突破之地，随着鄱阳湖生态经济区建设的持续实施，随着沿江开放开发战略的持续推进，随着重大项目积蓄的能量持续释放，我市赶超发展势头正劲，干事创业氛围正浓，仍处于加速发展、加快赶超的重要战略机遇期。我们必须牢牢把握“稳中求进，快中求好”的总基调，始终坚持加快发展不动摇，强工兴城不动摇，抓投资、攻项目不动摇，统筹发展不动摇，改善民生不动摇，努力在打造中部城市集群中抢占先机，在稳健增长、加速赶超中把握主动，在推进“四化同步”、全面建成小康中走在前列。

　　今年政府工作的总体要求是：全面贯彻落实党的十八大精神，高举中国特色社会主义伟大旗帜，坚持以邓小平理论、“三个代表”重要思想、科学发展观为指导，围绕九江争得应有地位、人民过上小康生活，加快推进鄱阳湖生态经济区建设，以沿江开放开发为龙头，以强工兴城为重点，以项目建设为抓手，打造"T"型工业带，构筑九江都市区，推进民生大改善，努力实现“五年全面进位”。

　　全市经济社会发展主要预期目标是：生产总值增长12%以上；固定资产投资增长25%以上；财政总收入增长20%以上；社会消费品零售总额增长15%以上；居民消费价格涨幅控制在3.5%左右；城镇居民人均可支配收入和农民人均纯收入分别增长12%、13%；人口自然增长率控制在8‰以内；全面完成节能减排任务。

　　实现上述目标，主要抓好以下九个方面工作：

　　（一）全力以赴战沿江，进一步加速龙头昂起

　　围绕实现“235”目标，举全市之力推进沿江开放开发，着力打造沿江港区、物流园区、工业园区和临港城区“四区一体”新格局，使之成为中部城市集群的战略支点。全年完成固定资产投资1200亿元、规模以上工业主营业务收入2700亿元。

　　夯实沿江平台强基础。加快港口基础设施建设，完善电子口岸平台，积极申报九江综合保税区。大力开拓国际航线，扩大上港码头集装箱转港接驳量，吞吐量超18万标箱；加快公共码头建设，港口货物吞吐量超5000万吨。加快物流园区建设，城西板块在做实做强上港物流园区和现代综合大市场的同时，预留1000亩以上集装箱物流园区用地；城东板块在做实做强新雪域物流园和大洪钢材市场的同时，预留1000亩以上中心城区生活性物流园区用地；赤码板块在加快推进中部庐山皮革红木大市场、九江（国际）皮草商贸城的同时，预留2000亩以上物流园区建设用地；彭湖板块在加快推进湖口轻工物流园、彭泽赣电物流园的同时，在定山预留2000亩以上物流园区用地。加快临港新城建设，以赤码板块的码头工业城二期和港口集镇，城东板块的临港、滨湖新城，城西港区二期，彭湖板块的海山、洋港新区和流泗、定山、马当为重点，建成一批功能齐全、配套完善、支撑有力的临港新城。加快疏港通道建设，启动建设城西港区铁路专用线，开展彭湖、赤码板块铁路专用线前期工作，建成赤码工业大道、彭湖工业大道（彭泽段），加强板块与疏港通道的衔接，构建立体快捷的集疏运网络。

　　壮大临港产业强支撑。根据沿江产业规划，以大运量、大用水、大进大出项目为重点，深入开展项目认证、审核、筛选、包装，不断充实完善沿江招商项目库，引进落户、推动建设一批产业项目，全力培育、重点打造一批临港产业集群。城西板块围绕“固定资产投资217亿元，主营业务收入570亿元”目标，重点引进汽车及零配件、新能源、新材料、电子电器等产业链项目，重点推进华祥科技、信源通等项目竣工投产；城东板块围绕“固定资产投资340亿元，主营业务收入800亿元”目标，重点引进电力能源、机械制造、精细化工、化纤玻纤、大宗粮油和绿色食品加工等产业链项目，重点推进恒生大化纤、青岛啤酒等项目竣工投产；赤码板块围绕“固定资产投资290亿元，主营业务收入610亿元”目标，重点引进新材料、装备制造、轻化纺织等产业链项目，重点推进理文化工、富美佳装饰新材料等项目竣工投产；彭湖板块围绕“固定资产投资360亿元，主营业务收入690亿元”目标，重点引进冶金、化工等产业链项目，重点加大神华煤电、千万吨级优质钢铁产业基地、南方水泥等项目推进力度。

　　用足用好政策强保障。加大政策争取力度，力争国家和省对沿江给予更多优惠政策，引导全省临港产业向沿江集聚；加强与省直部门衔接，全面落实财税、金融等各项支持政策。建立健全沿江开放开发规划执行、项目调度、岸线管理、协调服务、信息报送、目标考评、责任落实、投资融资等八项机制，形成统分结合、责任明确的工作格局。强化服务措施，加快组建运作沿江投资公司，破解资金难题；加强区域协作，启动沿江“飞地产业园区”建设，促进园区规划衔接、设施互通、信息互享、产业互补；支持赤码、彭湖板块申报省级经济开发区。

　　（二）坚定不移强工业，进一步扩张经济总量

　　继续加大强工力度，力争规模以上工业主营业务收入接近4000亿元、增加值突破700亿元、利税总额达到300亿元。

　　打造产业集群。以九江石化为龙头，启动石化产业园建设，积极引进上下游配套企业，加快推进石化油品质量升级工程、津晶城石化科技园等项目，着力打造千亿级石化产业集群；以九钢、江铜、厦钨等企业为重点，积极推进钢铁和有色金属深加工，延长产业链，打造千亿级钢铁和有色冶金产业集群；以九电、中电投等企业为重点，加快火电、风电等项目建设，打造千亿级电力能源产业集群；加大粘胶化纤和纺织服装产业承接力度，扩大产业规模，提升技术水平，打造千亿级粘胶化纤和纺织服装产业集群。加快电子信息、非金属新材料、新能源、节能电器、绿色食品等新兴产业发展，争取迅迪新能源、皓康光电等纳入省重点调度，扶持一批重点产业和骨干企业。围绕“十百千”目标，依托蓝星星火、卡博特等企业，力促有机硅单体产能达到70万吨，打造国家级有机硅产业基地；依托国家级恒盛科技企业孵化基地，着力引进30个以上科技孵化项目，打造国家级科技企业孵化基地；依托赛得利、龙达、恒生等化纤企业，力促粘胶纤维产能达到100万吨，打造国家级化纤产业基地；依托德鑫、锦兴、华孚、三鑫等企业，力促纺纱产能达到400万锭，打造国家级纺织产业基地；依托赛龙通信等企业，力促手机产能达到4000万台，打造省级手机产业基地。

　　做大骨干企业。通过推进技改扩能、重组合作、增资扩股，加快做大做强一批骨干企业，新增主营业务收入过亿元企业50户以上、过10亿元的5户以上，逐步把旭阳雷迪、赛龙通信、理文造纸、理文化工、攀森新材料、江铜铅锌冶炼、蓝星星火等企业打造成百亿企业。加大资金支持力度，扶持一批主业突出、成长性好、科技含量高的企业；全面落实支持小微企业发展的各项政策措施，鼓励中小企业主动承接骨干企业，引导骨干企业带动配套企业。强化企业扶持，出台优惠政策，引导外出务工人员返乡创业就业；协调银行和担保部门，帮助企业贷款融资，破解融资难题；市本级设立工业发展专项资金1亿元，用于鼓励企业技改扩能、技术创新、品牌创建、贷款贴息。

　　提升园区水平。按照园区产业定位，依托龙头企业，着力提升主导产业配套能力，促进园区产业集群发展；全面清理圈而不用、多占少用、违规改变性质的企业用地，实行供地与投资强度、产出效益等指标挂钩，引导园区由单一式供地向订单式厂房转变、由出让土地向租用土地和租售厂房转变；探索园区户籍管理、住房保障、社区建设、用工培训和企业文化建设新途径，加快园区金融、物流等服务业发展，提高园区服务社会化水平；加大生态园区建设力度，扩大园区绿化提升试点范围；建立健全园区经济运行调度办法，加强监测预测和分析调度。全年工业园区主营业务收入达2800亿元，实现工业园区个个过百亿。

　　（三）毫不动摇兴城市，进一步提升城市形象

　　按照五年基本建成八里湖新区的部署，中心城区实施城建项目200个，完成投资350亿元。全市城镇化率提高1.7个百分点。

　　着力打造大九江都市区。编制完成《大九江城市群空间发展战略规划》和《九江市城市综合交通体系规划》，健全完善“大九江”一体化体制机制，有效促进发展要素平等交换、公共资源均衡配置，有序推进农业转移人口市民化，逐步实现基本公共服务均等化。加快推进绕城高速、昌九高速（通远段）拓宽改造，开工建设通岭大道、威通线（南山段）、庐山机场路改造，撤销环庐山公路威家、隘口收费站，撤销昌九高速九江南、荷花垅、白水湖和九景高速新港收费站，新建昌九、九景高速九江主收费站，加快城市轻轨前期工作，促进“四县一山”与中心城区相向发展、融为一体。加快县城和中心集镇建设，充分发挥中心城区辐射带动作用，加大指导、帮扶和激励力度，在完善提升一批县城新区的同时，打造一批沿江特色产业集镇、沿路物流节点集镇、滨湖生态旅游集镇、山区边贸中心集镇。

　　基本建成八里湖新区。加大力度建项目，强功能，优环境，确保基础设施基本建成、城市功能基本完善、公共服务基本到位、开发项目全面铺开，努力实现“五年基本建成、人口接近10万”的目标。全面建成市民服务中心、文化艺术中心、市民中心公园、九龙山公园、八里诗词文化长廊、文博园、环湖四路及景观桥、南组团路网等项目；大力推进特色小镇、移动通信呼叫中心、中航城、水岸莲华等项目；开工建设国际金融广场、滨水商业街、兴城南大道、游船码头等项目。坚持市场运作、政策引导，鼓励部门进驻新城区，加快市委党校新校区、公安科技大楼、消防应急救援指挥中心、报业中心、总工会“三大场所”等项目建设步伐。依托资源和区位优势，包装开发一批水上游乐、旅游观光、商贸物流等项目，促进城市要素向新区集聚，逐步实现从建设城市新区向发展城市经济转变，着力打造生态新区、文明新区、美丽新区。

　　加快提升老城区。浔阳区以分片改造为重点，改造完成浔阳楼、战备路等片区，启动孔家洼、面粉厂等片区改造，加快建设花果园、琵琶亭等安置小区；加快推进长虹商务、繁都商务、九江烟草物流中心、京九市场搬迁改造等商贸项目，加快建成长虹东大道，改造提升白水湖公园，完善城市环卫、群众健身等公建设施。庐山区以整区改造为重点，加快五里片区和浔南城市客厅建设，全面完成欣荣路、濂溪大道延伸线等项目，继续推进宏大五金城、禧徕乐商贸中心、喜盈门国际广场、邹家河文化休闲街区等商贸项目，启动琴湖大道南段等项目建设。九江开发区以完善功能为重点，加快园区服务设施和大型城市综合体建设，推进联盛九龙广场、总部经济等项目，启动水果批发市场搬迁和三马路改造。

　　全面加强城市管理。以城市创建为载体，大力开展违章占道、违法建设、建筑工地、交通秩序、垃圾广告等专项整治行动，继续整治“两声三乱”，开展城管进学校、进社区活动，不断净化美化城市环境，争创省级生态园林城市，启动国家历史文化名城和森林城市创建。以科学管理为抓手，加快数字城管指挥中心建设，提高城管信息化水平。加快东郊垃圾处理场和第二垃圾处理场配套项目建设，确保中心城区垃圾无害化处理率100%。以优化水系为重点，按照“大水系连通、水环境改善、水安全保障、水生态良好”的目标，加快推进浔南、濂溪片区截污工程和白水湖污水处理厂、十里河补水工程，改善中心城区水生态环境。

　　（四)强化措施抓“三农”，进一步夯实农业基础

　　坚持“三农”基础地位不动摇，着力促进农业现代化与工业化、信息化、城镇化同步发展，逐步形成以工促农、以城带乡、工农互惠、城乡一体的发展格局。

　　发展现代农业。加快农业规模化，强化土地流转的政策支持和服务引导，有序推进土地规模经营，促进规模开发、连片开发，打造10个万亩、100个千亩现代农业示范区，争创一批国家级现代农业示范区；培育一批苗木基地，启动市花卉交易中心建设。加快农业产业化，新引进3000万元以上项目70个，新增规模以上农业企业20家，加快推进神州通油茶等项目建设，支持鸭鸭、博莱、仙客来、绿冬丝科等企业做大做强；建成8个农产品加工园区，创建20家省级农民专业合作示范社；加快病险水库除险加固、小农水重点县等水利工程建设，恢复和新增灌溉面积9万亩，改善灌溉面积18万亩，建设高标准农田6万亩，提高农业综合生产能力。加快农业机械化，加大农机推广力度，提高机耕、机种、机收等现代农业装备应用水平；启动市农科院组建工作，提高农业科技成果转化率。加快农业标准化，支持鼓励农产品商标注册，加大“三品一标”申报认证力度，新增无公害农产品、绿色食品、有机食品和地理标志农产品50个以上。

　　改善农村面貌。扎实推进和谐秀美乡村建设，市本级财政投入资金4600万元，完成800个新农村建设点村庄整治，改善农村水、电、路等基础设施，打造一批种养、加工、餐饮、休闲养老等专业村庄，建设农村信息化乡镇30个、信息服务站300个。编制完成乡镇、村庄规划，推进农房改造，加强建房管理，推广特色房型。大力整治“两违”建筑，全面完成“两沿”村庄整治提升任务。按照“清洁家园、清洁水源、清洁田园、清洁能源”的要求，加强农村生活污染综合治理，深入实施农村清洁工程，生活垃圾无害化处理率提高10个百分点。鼓励发展村级经济合作组织，开发集体资金、资产、资源，发展壮大村级集体经济。创新和完善农村矛盾调处机制，切实解决好农村留守儿童和老人的实际问题。

　　促进农民增收。继续稳定粮、棉、油等大宗农产品生产，培育壮大水产、茶叶等特色产业。大力发展现代化经营方式和组织形式，加快推进种养加、产供销、贸工农一体化经营。完善“龙头企业+专业合作社+农户”等利益联结机制，增强产业带动力。实施好“阳光工程”、“雨露计划”和“一村一名大学生”工程，完成星火科技培训8万人，造就一批新型农业农村人才。全面落实强农惠农政策，坚决纠正损害农民利益的突出问题。

　　（五）攻坚克难抓项目，进一步增强发展后劲

　　围绕“固定资产投资突破1600亿元”的目标，坚持重大项目带动，全面聚焦项目，全力引进项目，全程服务项目，全速推进项目。

　　强化举措上项目。对接国家和省重大投资动向及重点投资领域，争取一批基础设施和重大产业项目；对接鄱阳湖生态经济区建设，争取一批生态和新兴产业项目；对接老工业基地调整改造等支持政策，争取一批技改和扩能项目。强化高端招商、专业招商、以商招商和活动招商，切实提高招商专业性和针对性，全年实际利用外资9亿美元以上，引进内资430亿元以上，实现外贸出口30亿美元以上，利用外资超1000万美元项目10个以上，外贸实绩企业300家以上。

　　精心操作推项目。加快推进九宏新材料、宏狄氯碱等一批重大产业项目；加快推进九江长江公路大桥、赛城湖跨湖大桥、昌九高速（永修至共青段）改扩建、修平高速、武九客专、合九客专、九景衢铁路、都九高速公路、罗石公路（武宁罗溪至靖安石境）等一批重大交通设施项目；加快推进婺桃线、立肇线、彭乐线等一批区间公路项目；加快推进南昌至湖口二级航道整治等一批港口配套项目；加快推进九电四期、石钟山至景德镇洪源线路、码头工业城和德安县工业园220千伏输变电、德化110千伏输变电等一批重大电力能源项目；加快推进青少年科普教育基地、庐山西海演艺中心、庐山古镇等一批文化旅游项目；加快推进九江学院八里湖校区和九江职业大学、九江职业技术学院、江西财经职业学院新校区等一批社会事业项目。

　　优化环境帮项目。继续实行“六个一”项目推进机制，进一步强化项目责任、调度和督查，及时研究解决项目推进中的困难和问题。大力整治“中梗阻”和“乱作为”，继续健全行政审批“一站式”服务、投资项目审批服务代办、重大工程项目跟踪服务等制度。继续加大要素保障力度，积极争地清地，集约节约用地；大力争资融资，充分激活民资；加快引才引智，优化用工环境；科学调度调配，保障煤电油运气。

　　（六）统筹兼顾促协调，进一步激发赶超活力

　　壮大县域经济。强化分类指导，促进沿江各地放大核心优势，抢抓发展机遇，做大临港产业，加快联动发展，着力打 造沿江产业带；促进沿路各地放大区位优势，突出产业特色，做大主导产业，加快推进共青城与德安、永修与南昌桑海、九江县与九江开发区相向发展，着力打造工业大走廊；促进环山各地放大旅游优势，依托庐山龙头，建设精品景区，加快融合发展，着力打造旅游大品牌；促进山区湖区放大资源优势，发展矿业经济，推动返乡创业，加快内生发展，着力打造新的增长点。支持共青城申报国家级高新技术开发区、打造全面建成小康社会先导区，支持星子创建全国旅游标准化示范县，支持武宁、修水、都昌等地发展矿业经济。健全和完善考核、调度、激励机制及县域经济考核评价体系，建立市县联动和县域协调机制，促进竞相发展。实现县县财政过10亿元， 4个过20亿元。发展文化旅游。以庐山行政服务中心下迁为契机，进一步强化中心城区旅游集散功能，加强庐山与中心城区、星子、庐山西海、共青城在品牌宣传、线路整合、旅游促销等方面的 联动，深化山上山下融合发展。加强景区基础设施建设，完善提升购物、餐饮、休闲、娱乐等服务配套。

　　加快旅游资源开发，推进中信庐山西海度假区、庐山西海休闲欢乐世界、武宁国际休闲养生度假区、修水温泉旅游度假区、庐山天然气管道等项目建设，支持创建4A级“山水武宁”景区；加强文化遗产保护，挖掘非遗资源和民俗文化；发展文化创意、数字动漫、演艺娱乐、广告会展等产业，培育新兴文化旅游业态。强化宣传促销，继续在央视开展九江旅游整体形象宣传，拓展旅游专列、游轮客源市场；积极开拓境外市场，巩固提升传统市场。全年接待旅游总人数、旅游总收入增长15%以上。

　　繁荣商贸物流。修编沿江物流、环鄱阳湖码头和商贸物流发展规划，完善中心城区和县城商业网点规划，加快构建“城内大商场、城郊大市场、城乡大流通”的发展格局，逐步打造区域性现代商贸物流中心。大力推进“6620”商贸重点工程建设，继续推进“万村千乡市场工程”，进一步完善农村商贸流通网络。加大资源整合力度，完善物流服务体系，提升成熟商圈品质，健全新兴商圈功能，加快构建综合运输、口岸通关、信息网络、政策环境等服务平台，提升现代物流业发展水平。

　　（七）加大力度优生态，进一步转变发展方式

　　加强生态建养。更加注重生态文明建设，倾力打造天蓝、地绿、水净的美好家园。加强湖泊保护与管理，保护好鄱阳湖“一湖清水”。加强水源、土地、森林、湿地和野生动物等资源保护，治理水土流失24万亩，创建7个省级生态乡镇、7个省级生态村。巩固造林绿化成果。从今年起，市本级财政每年安排3000万元，整合资金3亿元，推进“森林城乡、绿色通道”建设。启动市区PM2.5监测工作，加快县级环境监测站标准化建设，加大企业超标排污整治和处罚力度，强化环境应急防范措施，坚决打击企业故意排污和恶性偷排行为，重点解决损害群众健康的突出问题，切实维护资源环境安全。加强生态文明宣传教育，增强群众的节约、环保和生态意识，形成合理消费、爱护生态的社会风尚。

　　发展低碳经济。严格执行能耗限额标准，加强减排项目管理，逐步淘汰落后工艺和设备。降低能源、水、土地消耗强度，切实提高资源利用效率和效益。加强企业间产品的循环利用，形成副产品、能源、废弃物和原材料相互交换的工业生产系统。支持九江出口加工区争创国家级循环经济示范园区，支持武宁打造生态文明试验示范区。抓好金砂湾工业园、云山经济开发区循环经济试点工作，积极探索循环经济发展新模式，促进生产、流通、消费过程的减量化、再利用、资源化。

　　推进科技创新。牢牢把握转变发展方式的主线，实施创新驱动发展战略，大力推进科技协同创新。安排专项资金1900万元支持科技事业发展，确保科技经费不低于市本级财政支出的1%。依托优势资源和特色产业，积极引进高科技、高投入、高效益项目，抢占产业制高点，增强核心竞争力。突出企业主体，建立健全创新型企业培育机制和企业牵头实施重大项目机制，积极组建协同技术创新中心和产业技术创新联盟，支持江联造船、巨石集团、中星医药等企业申报省级技术中心。深化科技、人才、项目对接，鼓励企业建设研发机构、引进高端人才，支持企业与高校、科研院所开展科技合作。深化科技管理体制改革，发挥政策支持和财税引导作用，建立市本级科技创新综合服务平台，鼓励发展科技中介服务机构。新增国家级高新技术企业15家，新建省级以上科技园区和产业基地5个，开发省级重点新产品40个以上，完成科技成果转化项目100个，专利申请1000件以上，科技进步贡献率达50%以上。

　　（八）千方百计惠民生，进一步增进人民福祉

　　坚持把保障和改善民生作为政府工作的出发点和落脚点，不断加大民生投入，让全市人民共享赶超发展的成果。

　　全面加快建成小康社会步伐。编制完成全面建成小康社会实施纲要，细化指标体系，分解目标任务，健全工作机制，明确实施步骤，强化保障措施，加大投入力度，努力为人民群众谋取更好的教育、更稳定的工作、更满意的收入、更可靠的社会保障、更高水平的医疗卫生服务、更舒适的居住条件、更优美的环境。

　　突出抓好民生工程。财政支出民生资金180亿元以上，在全面完成省下达民生工程任务的基础上，围绕保障性住房建设、就业创业、社会保障、公共服务、改善人民群众基本生活条件等方面，实施好十项惠民工程：

　　1.实施安居工程：建设保障性住房10万套以上，改造各类棚户区3.7万套以上，新建公租房6.3万套以上，其中中心城区新建4万套；实施农网“低电压”整治和智能电表改造；完成难点难户供水一户一表安装改造9000户；新增管道天然气用户5000户；新建农村户用沼气8000座。

　　2.实施乐业工程：健全就业帮扶长效机制，完善职业培训政策措施。新增发放小额贷款4亿元；新增城镇就业5.5万人，新增转移农村劳动力5.3万人；为城乡困难群众购买公益性就业岗位4400个。

　　3.实施温暖工程：城市低保标准提高到400元，人均月补差金额提高到240元；农村低保标准提高到200元，人均月补差金额提高到120元；孤儿机构抚养标准每月1000元以上；农村五保户集中、分散供养标准分别提高到2880元、2760元，城镇“三无”特困群众月人均供养标准提高到450元；开工建设市社会养老福利中心和救灾仓库，推进庐山西海国际养生养老中心建设；完善残疾人和流浪未成年人救助保障机制；加强“三院”建设和管理；对城区1万户低收入家庭发放价格补贴。

　　4.实施菜篮子工程：新扩城郊蔬菜基地6000亩；完善提高20家农超对接网点；完成中心城区4家农贸市场升级改造，中心城区新建20家社区便民菜店。

　　5.实施畅通工程：坚持公交优先，加强客运市场监管，加快推进公交场站建设，增加通往保障性安居小区、八里湖新区公交班次和线路，中心城区新增公交车30辆；改造交通瓶颈，在中心城区主要路口增设一批智能交通控制设施；清理规范现有停车资源，在中心城区新建2个公共停车场；整治城区边街小巷20条以上，改造人行道1万平方米；完成农村公路建设800公里。

　　6.实施安康工程：完善农村三级卫生服务网络、城镇医疗卫生服务体系和社区卫生服务体系，招聘卫生技术人员600名；实行免费孕前优生健康检查；启动贫困家庭重性精神病患者免费救治；完成红十字应急救护培训1.2万人；启动市食品药品检验大楼建设，开展“放心粮油”、“放心食品”整治活动；解决25万农村人口饮水安全问题。

　　7.实施文化惠民工程：建设市级“数字图书馆”；新建和改造光纤入户小区300个，覆盖家庭10万户；建设140个行政村农民体育健身场所。

　　8.实施扶贫开发工程：实施395个扶贫开发重点村整村推进和水库移民后期扶持项目；完成深山区、地质灾害区、环境污染区移民搬迁9000人以上。

　　9.实施防灾减灾工程：建设市地震台网中心；建立气象双向预警信息传递机制，在农村重点区域建设雷电观测网；建设农村气象灾害防御体系和农业气象服务体系，提高灾害防御能力。

　　10.实施平安工程：深化平安建设，打造平安九江，完善立体化社会治安防控体系；启动“天网”三期工程，建成庐山大道、十里大道、九瑞大道、长江大桥公安检查站视频监控系统，中心城区新增监控探头500个，实现城区公共部位视频监控全覆盖。

　　加强和创新社会管理。建立健全“党委领导、政府负责、社会协同、公众参与、法治保障”的社会管理体制，推动社会管理重心下移。严格落实《信访条例》，畅通和规范群众诉求表达、利益协调、权益保障渠道，完善人民调解、行政调解、司法调解联动体系，加大信访积案和矛盾纠纷排查调处力度。建立健全新闻舆论引导、监管和应对机制，加强网络文化建设与管理，提高对热点问题和突发事件的快速反应能力。严格落实安全生产责任制和事故责任追究制，深入开展安全隐患专项治理，全面推进职业健康安全监管，着力保障道路交通、矿山、建筑工地、危化物品、校园校车等重点领域安全。全面加强社会管理综合治理，突出彰显人文关怀，加强和创新流动人口和特殊人群管理服务，强化司法基本保障，做好刑释解教人员安置帮教工作，有效预防青少年违法犯罪，依法防范和惩治违法犯罪活动，切实保障人民群众生命财产安全。

　　繁荣发展社会事业。进一步加大教育投入，全面完成学前教育三年行动计划，继续实施“两免一补”政策，加快发展职业教育和高等教育，全面提升教育质量。落实国家基本药物制度，抓好卫生信息化和卫生防疫工作，提高无偿献血占临床用血比例。加快推进九江学院附属医院门诊综合大楼、市三医院肿瘤内科综合大楼、市五医院住院综合大楼、市儿童医院等项目建设。加强食品药品安全监管，继续推进全国食品药品安全示范区创建工作。分类推进事业单位改革，深入推进教育、文化、医药卫生和财税、投融资等体制改革；稳步推进国资监管全覆盖，做实做强投融资平台，确保国有资产保值增值。认真做好第三次全国经济普查工作。严格落实计划生育政策，大力提高出生人口素质，创新完善流动人口计划生育管理服务。支持驻浔部队和国防后备力量建设，加强国防宣传教育，深入开展双拥共建，促进军地融合式发展。抓好民族宗教、外事侨务和对台工作，促进物价、审计、工商、质监、海关、检验检疫、人民防空、盐业、邮政、水文、地方志、档案、保密、编制、无线电管理、工会、共青团、妇女儿童、老龄、残疾人、红十字和慈善等社会事业和谐发展。

　　（九）不遗余力提效能，进一步加强政府自身建设

　　按照“干部清正、政府清廉、政治清明”的要求，全面落实中央改进工作作风、密切联系群众八项规定，全力建设职能科学、结构优化、廉洁高效、人民满意的服务型政府。

　　坚持依法行政。坚决贯彻市委的决策部署，自觉接受市人大及其常委会的法律监督和市政协的民主监督，广泛听取各民主党派、工商联和无党派人士的意见建议，主动接受媒体监督和社会公众监督，认真办理人大代表议案、建议和政协提案。牢固树立社会主义法治理念，严格按照法定权限和程序行使权力、履行职责，切实做到严格、规范、公正、文明执法。认真执行民主集中制，继续实行重大项目和重大决策风险评估机制，建立健全决策问责和纠错制度，加强经济责任审计，推进权力运行公开化、规范化，让人民监督权力，让权力在阳光下运行。

　　坚持为民执政。坚持把人民利益放在第一位，重大决策顺民而定，工作措施应民而出，发展成果由民共享。以开展“为民务实清廉”主题教育实践活动为契机，改进调查研究，多到困难、矛盾集中和群众意见多的地方，问政于民，问需于民，问计于民，全力把矛盾解决好，把民生改善好。坚决克服形式主义、官僚主义，坚决纠正行政不作为、乱作为和损害群众利益的不正之风，着力解决人民群众反映强烈的突出问题。

　　坚持高效理政。继续实行简政放权，规范行政审批程序，切实改进会风文风，加强行政机关信息化建设，不断提高行政效能。继续保持求真务实、埋头苦干的工作作风，做到不畏难、不言难、不避难，知责担当，强化操作，攻坚破难。继续完善评价考核指标体系，加大重点工作督查督办力度，做到令行禁止，确保政令畅通。继续开展政风行风民主评议活动，着力整治庸懒散奢等不良风气，努力打造一支政治坚定、能力过硬、作风优良、奋发有为的公务员队伍，着力提高政府的公信力、执行力和操作力。

　　坚持清廉从政。按照“标本兼治、综合治理、惩防并举、注重预防”的方针，认真贯彻落实党风廉政建设责任制和廉洁自律的各项规定，加强廉政教育，深化廉能管理，防范廉政风险。认真抓好工程建设、政府采购、资金使用、公共资源和产权交易等重点领域和重要环节的监管，严肃查处以权谋私、贪污贿赂、失职渎职、损害群众利益、破坏发展环境等违纪违法行为。牢固树立过紧日子的思想，加强收支预算管理，厉行节约，勤俭办事，把有限的资金更多用于惠民生、促发展上。

　　各位代表，奋起赶超奠定坚实基础，应有地位昭示光荣使命，小康生活承载人民期盼。让我们在市委的坚强领导下，以党的十八大精神为指引，坚持目标不动摇、路径不折腾、重点不摇摆，鼓足干劲，锐意进取，全力实现“五年全面进位”，为“九江争得应有地位，人民过上小康生活”而努力奋斗！
